# The Treatment of Pyosalpinx

**Published:** 1890-03

**Authors:** 


### Editorial.

### THE TREATMENT OF PYOSALPINX.

The discussion upon this subject which recently took place in the Philadelphia Obstetrical Society, and which is reported in this issue of the JOURNAL, is an exceedingly interesting one, since it ably presents the two sides of the subject as viewed by the advocates of widely differing opinions. On the one hand the so-called "tube men" claim that recurrent pelvic inflammations,—the pelvic cellulitis of Emmet—are significant of serious disease in the Fallopian tubes, whose persistence is so detrimental to health and so dangerous to life as to call imperatively for the removal of the diseased organs by laparotomy. This view is sustained by a pathology of the disease, which is undoubtedly correct, since the operator is able to exhibit the tubes in a state of disease and distended with pus. To emphasize this point a "bucketful of pus tubes" was exhibited by a single operator at the meeting referred to. A further argument is drawn from the surgical principle that the presence of pus is not to be tolerated in any part of the body. To clinch the argument it is asserted, and undoubtedly with truth, that patients who have long been invalids are restored to health by the operation. Hence the conclusion is reached that when it can be demonstrated that the tubes are distended with fluid, the only course open to the physician is to remove such tubes.

But many gynecologists of equal repute and equal skill fail to accept these extreme views. Arguing from a clinical standpoint, they claim that pus in the Fallopian tubes differs from collections of pus in most other parts of the body, in the fact that it is simply enclosed within the cavity of an organ, and is not located within the tissues themselves. They further claim that many cases of undoubted pyosalpinx end in recovery without an operation, under judicious treatment, the pus being discharged from the tube through the uterus or becoming inspissated and reduced to a cheesy condition in which it may remain perfectly innocuous for an indefinite length of time. It is even claimed and verified by the citation of cases, that women who have been the subject of pyosalpinx have been so far restored to health without operation as to subsequently bear children.

Between these two views the choice which is made by any individual will depend very largely upon the mental characteristics of the chooser. If he be surgically inclined and fond of the brilliancy and *eclat* of capital operations he will be apt to decide that laparotomy is the preferable course to pursue. To cure a woman in three or four weeks by a successful operation of an ailment which has existed for months or years, and to be able to exhibit the trophies and report the results to his brother physicians offers a strong temptation to the ambitious surgeon. But if he be imbued with the somewhat obsolete idea of the sanctity of the human body, and with a reluctance to mutilate organs which can be preserved intact, he will be apt to prefer the less brilliant, but more conservative procedures, which will give an opportunity for the restoration of the parts to their normal conditions and normal functions. It is unquestionably true that in such efforts he will often fail in the treatment of pyosalpinx. But when as a last resort the removal of the tubes is undertaken, it will be done with the consciousness that the organs are condemned only after they have been proven to be hopelessly incurable.

There is no doubt that as time goes by further experience will tend to reconcile these wide differences of opinion between earnest workers in the same field. Meanwhile we would suggest that dogmatic assertions and ridicule of opposing views will not help to bring about this desirable end. It is only by long-continued observations and comparison of results that the truth will be finally reached. And it will probably be found in the end, as in most cases of difference of opinion between honest men, that the truth lies at a point midway between the two extremes.

---

### ITEMS.

INVITATION TO THE TENTH INTERNATIONAL MEDICAL CONGRESS.—In accordance with the decision of the Ninth Congress at Washington, the Tenth International Medical Congress will be held at Berlin from the 4th to the 9th of August, 1890.

By the delegates of the German Medical Faculties and the chief Medical Societies of the German Empire, the undersigned have been appointed members of the General Committee of Organization. A Special Committee of Organization has also been appointed for each of the different sections, to arrange the scientific problems to be discussed at the meetings of the respective sections. An International Medical and Scientific Exhibition will also be held by the Congress.

We have the honor to inform you of the above decisions, and at the same time cordially to invite your attendance at the Congress. We should esteem it a favor if you would kindly extend this invitation to your friends in Medical Circles, as way may offer.

DR. RUDOLPH VIRCHOW,
President.

DR. VON BERGMANN, DR. LEYDEN, DR. WALDEYER,
Vice-Presidents.

DR. LASSAR,
E. Secretary General.

All communications must be directed to the General Secretary, Berlin NW., Karlstrasse 19.

